# Design and In Silico Validation of a Novel MZF-1-Based Multi-Epitope Vaccine to Combat Metastatic Triple Negative Breast Cancer

**DOI:** 10.3390/vaccines11030577

**Published:** 2023-03-02

**Authors:** HemaNandini Rajendran Krishnamoorthy, Ramanathan Karuppasamy

**Affiliations:** Department of Biotechnology, School of Bio Sciences and Technology, Vellore Institute of Technology, Vellore 632014, Tamil Nadu, India

**Keywords:** myeloid zinc finger 1, triple-negative breast cancer, immunoinformatics, vaccine, immunotherapy, docking, dynamics simulations

## Abstract

Immunotherapy is emerging as a potential therapeutic strategy for triple negative breast cancer (TNBC) owing to the immunogenic landscape of its tumor microenvironment. Interestingly, peptide-based cancer vaccines have garnered a lot of attention as one of the most promising cancer immunotherapy regimens. Thus, the present study intended to design a novel, efficacious peptide-based vaccine against TNBC targeting myeloid zinc finger 1 (MZF1), a transcription factor that has been described as an oncogenic inducer of TNBC metastasis. Initially, the antigenic peptides from MZF1 were identified and evaluated based on their likelihood to induce immunological responses. The promiscuous epitopes were then combined using a suitable adjuvant (50S ribosomal L7/L12 protein) and linkers (AAY, GPGPG, KK, and EAAAK) to reduce junctional immunogenicity. Furthermore, docking and dynamics analyses against TLR-4 and TLR-9 were carried out to understand more about their structural stability and integrity. Finally, the constructed vaccine was subjected to in silico cloning and immune simulation studies. Overall, the findings imply that the designed chimeric vaccine could induce strong humoral and cellular immune responses in the desired organism. In light of these findings, the final multi-epitope vaccine could be used as an effective prophylactic treatment for TNBC and may pave the way for future research.

## 1. Introduction

Triple-negative breast cancer (TNBC) is described as highly heterogeneous due to the non-existence of canonical targets such as the estrogen receptor (ER), human epidermal growth factor receptor 2 (HER2), and the progesterone receptor [1]. For decades, the therapeutic options for TNBC have been limited to conventional strategies such as chemotherapy, surgery, and radiotherapy. Nonetheless, these strategies have downsides, such as substantial toxicity and poor efficacy [2,3]. In addition, patients with TNBC also exhibit resistance to hormonal and targeted treatments due to genetic changes in metastatic tumor cells [4]. Thus, considering the invasiveness, drug resistance, and heterogeneity, there is an unmet need for novel therapeutic approaches for the management of TNBC.

Recent advancements in cancer immunotherapy, which triggers the host immune system to eliminate cancer, are now revolutionizing the therapeutic landscape of TNBC [5]. Intriguingly, TNBC has a high immunogenic tumor microenvironment associated with elevated levels of tumor-infiltrating lymphocytes (TIL), tumor-associated macrophages (TAMs), and mutational load, which are related to higher responsiveness to immunotherapy [6]. Amongst various immunotherapy strategies, the use of subunit or peptide-based vaccines to treat cancer is considered intriguing because of their potential to stimulate a targeted immune response with minimal toxicity [7]. It is noteworthy that 45.5% of breast cancer vaccines undergoing clinical trials are peptide-based. For instance, some of the subunit vaccines, namely TPIV200 (NCT02593227), PVX-410 (NCT04634747), and P10s-PADRE (NCT02938442), are currently in clinical trials to combat TNBC [8]. Moreover, in-depth research is also being conducted to recognize probable peptide vaccines for the treatment of TNBC. Studies from the literature show that a mixed 19-peptide vaccine alone improved prognosis of patients with refractory TNBC [9]. Similarly, Abdelmoneim et al. (2021) used matrix metalloproteinase-9 (MMP9), an essential protein for the survival and metastasis of solid tumors, as a target to design a universal cancer vaccine [10]. To date, the most widely explored tumor antigens include mucin-1 (MUC-1), carcino-embryonic antigen (CEA), HER2, and cancer-testis antigens. However, there is a substantial percentage of differentially expressed proteins that provide hope as potential vaccine candidates.

Dysregulation of transcription factors (TFs) represents a distinct class of drug targets as it facilitates cancer initiation, progression, and metastasis. Hence, focusing on these mutated transcription factors may result in new therapeutic strategies [11]. From the therapeutic viewpoint, preventing interactions among TFs poses a daunting issue since the contact areas of TFs are comparatively wide and featureless. However, effective tools have surfaced to address the targeting issue. According to the literature, myeloid zinc finger 1 (MZF-1), a member of the Krüppel family of zinc fingers, is reported to be overexpressed and is connected to cell invasion, mesenchymal phenotype, and migration in TNBC. Importantly, it has been proposed that MZF-1 could serve as a therapeutic option with promise for the management of TNBCs that express MZF-1 at high levels. Though MZF-1 plays a significant role in TNBC progression, relatively little research has been done with it [12].

Recently, new breakthroughs in computational techniques for identifying antigenic epitopes and developing subunit vaccines have drawn a lot of attention. Interestingly, the cost-effective, highly specific, and time-saving immunoinformatic approaches have already helped researchers to predict potential antigenic epitopes required for the development of a multi-epitope vaccine candidate [13]. Furthermore, there is a multitude of work being done to design TAA-specific cancer vaccines via immunoinformatics strategy. For instance, Khairkhah et al. (2022) employed an in silico immunoinformatics strategy to identify potential CTL-, HTL-, and B-cell-inducing epitopes against SARS-CoV-2 based on S, N, and M proteins. It is worth mentioning that the designed vaccine significantly stimulated total IgG, IgG2a, IFN-γ, TNF-α, IL-15, IL-21, IL-6, and Granzyme B secretion under in vitro and in vivo conditions [14,15]. In another study, Akbari et al. (2021) utilized a similar kind of pipeline to design two multi-epitope fusion constructs based on HIV-1 and Hsp70 [16]. Similarly, Jahangirian et al. (2022) used immunoinformatics tools to identify potential CTL and HTL epitopes from PRAM, BAGE4, and BLCAP antigens to construct a novel multi-epitope vaccine against bladder cancer [17]. In another study, Kumar et al. (2022) incorporated the epitopes from L1, E5, E6, and E7 oncoproteins of HPV 16 and HPV 18 to formulate a chimeric peptide-based vaccine to combat cervical cancer [18]. Dar et al. (2022) implemented a reverse vaccinology approach to determine the immunogenic B, CTL, and HTL epitopes from NT5E, ANPEP, and MME proteins, the putative targets for gallbladder cancer (GBC), and developed a robust peptide-based vaccine against GBC [19]. Importantly, our team successfully developed an immunoinformatics-based peptide vaccine targeting SOX9, a transcription factor that has been reported as a major regulator of TNBC metastasis [20]. With these frames of reference, the present study intended to develop a subunit vaccine integrating immunoinformatics methods and simulation studies targeting MZF-1, a recently reported oncogenic inducer, pertaining to the treatment of TNBC patients.

## 2. Materials and Methods

### 2.1. Retrieval of MZF-1 Protein

The amino acid sequence of the myeloid zinc finger 1 (MZF-1) protein (Accession no. P28698) was obtained in FASTA format from the UniProt (https://www.uniprot.org/) (accessed on 12 February 2023) database.

### 2.2. Prediction and Prioritization of Immunogenic Epitopes

The interaction of antigenic peptides with MHC molecules is an essential step in stimulating protective cellular immunity. Therefore, to identify the potential epitopes that bind to MHC class I and class II molecules, the ANN-based web servers NetMHCpan-4.1 (https://services.healthtech.dtu.dk/service.php?NetMHCpan-4.1) (accessed on 12 February 2023) and NetMHCIIpan-4.0 (https://services.healthtech.dtu.dk/service.php?NetMHCIIpan-4.0) (accessed on 12 February 2023) were used with the default parameters [21]. In parallel, to stimulate humoral immunity, B cell epitopes which interact with antibodies were recognized using the ABCPred, a recurrent-neural-network-based web server with default specification including window length of 16 mer at a threshold of 0.5 [22].

Furthermore, the potent immunogenic peptides were narrowed down based on their antigenicity, allergenicity, ability to induce IFN-γ, and toxicity. The antigenic potential of the peptides was computed using Vaxijen, an alignment-independent-based server that detects protective antigens at a 0.5 threshold for tumor models with 85% accuracy [23]. Similarly, with the default options set, AllerTop v2.0 and ToxinPred were used to assess the allergenicity and toxicity of the derived epitopes [24,25]. Additionally, the MHC II epitopes were sorted by their ability to provoke IFN-γ, which is vital for activating macrophages and natural killer cells [26]. The IFN-epitope server with the support vector machine (SVM) and a motif hybrid method was utilized to assess the IFN-γ-inducing peptides [27].

### 2.3. Population Coverage Analysis and Multi-Epitope Vaccine Formulation

The hurdle in engineering peptide-based vaccines is HLA heterogeneity. Therefore, the proposed vaccine ought to cover a large portion of the population. Accordingly, the global population coverage of the generated epitopes was explored using the Immune Epitope Database (IEDB) population coverage analysis tool [28]. Furthermore, the final chimeric vaccine was modelled using the chosen CD4+, CD8+, and B cell epitopes. To minimize junctional immunogenicity and to strengthen the immune response, KK, AAY, GPGPG, and EAAAK were used as linker molecules. In the past few decades, different toll-like receptor (TLR) agonists have been shown to be effective vaccine adjuvants, and they are currently being tested in clinical trials for their competency to orchestrate anticancer immunity. Thus, in this study, the 50S ribosomal L7/L12 protein (Accession No.: P9WHE3), a TLR-4 agonist, was used as an adjuvant molecule to boost a robust immune response.

### 2.4. Evaluation of Physicochemical Characteristics of the Vaccine

During vaccine design, understanding the essential attributes required for evoking a protective immune response is of great importance [29]. Hence, the immunological characteristics such as antigenicity, toxicity, and allergenicity of the vaccine were evaluated via the Vaxijen v2.0 (http://www.jenner.ac.uk/VaxiJen) (accessed on 12 February 2023), ToxinPred (https://webs.iiitd.edu.in/raghava/toxinpred/algo.php) (accessed on 12 February 2023), and AllerTop v2.0 (http://www.ddg-pharmfac.net/AllerTOP) (accessed on 12 February 2023) servers, respectively. Furthermore, using the Expasy ProtParam server, the physical and chemical properties including the aliphatic index, GRAVY (Grand average of hydropathicity index), molecular weight, instability index, theoretical isoelectric point (pI), and amino acid composition were obtained for the final designed vaccine [30].

### 2.5. Modelling and Validation of Secondary and Tertiary Structure

The secondary structure of the protein has an impact on the folding and three-dimensional shape of the vaccine. Therefore, in the current study, the secondary structural characteristics such as the β-sheet, alpha-helix, and turns of amino acid sequences of the constructed vaccine were anticipated through PSI-blast-based secondary structure prediction (PSIPRED), which incorporates two feed-forward neural networks to perform analysis obtained from PSI-BLAST (Position Specific Iterated BLAST) [31].

Similarly, the tertiary structure of the constructed vaccine was determined using the ROBETTA server and the structures were validated using the Ramachandran plot obtained from PROCHECK and quality score from the ProSA and ERRAT servers, respectively [32].

### 2.6. Analysis of Discontinuous B Cell Epitopes

The structural folding of a protein may lead to the formation of new discontinuous B cell epitopes, necessitating further predictions. Therefore, the Ellipro server was used to compute the conformational B cell epitopes. The server visualizes a protein’s 3D structure as an ellipsoid and assigns a protrusion index (PI) score to each predicted conformational epitope based on the mass of the residues that are outside the greatest possible ellipsoid. In addition, the PI score is proportional to the solvent accessibility of the residues; the higher the PI score, the higher the solvent accessibility of the residues [33].

### 2.7. Molecular Docking Analysis

Molecular docking is a significant technique to determine the predominant interactions between a ligand and a protein of known structure [34]. Therefore, the designed vaccine was docked against various TLRs including TLR-2, 4, 7, and 9 by the ClusPro 2.0 (https://cluspro.bu.edu/login.php) (accessed on 12 February 2023), a fully automated algorithm that performs a rigid-body protein–protein docking. It refines the docked poses that have strong surface complementarity and ranks them according to the clustering quality [35]. Finally, the bound complexes were visualized using the PyMOL software, and the interactions between the TLRs and vaccine were anticipated to provide insight into their binding scheme.

### 2.8. Molecular Dynamics Simulation Analysis

The GROMACS v2020.1 with GROMOS96 54a7 forcefield was employed for the molecular dynamics simulation study. Initially, the editconf inbuilt tool was used to compose a 10 Å dodecahedron box. Furthermore, the system was solvated and neutralized, respectively, by adding SPC water molecules and 14 sodium counter ions using the genion tool. Following this, the energy minimization of the complex was performed using the Steepest Descent algorithm. The van der Waals interactions and hydrogen bonds were constrained by using the LINCS algorithm. Subsequently, the isothermal and isobaric ensembles was employed for a time frame of 50 ps and the system was heated to a constant temperature and pressure of 300 K (optimal physiological temperature) and 1 bar pressure, respectively, using the Berendsen algorithm. Finally, the vaccine-TLR (TLR-4 and TLR-9) complexes were submitted to 30 ns MD simulations. The variables, such as radius of gyration (Rg), solvent-accessible surface area (SASA), and root-mean-square-deviation (RMSD), were computed utilizing the GROMACS utilities.

### 2.9. Codon Adaptation and In Silico Cloning

Back translation and codon optimization were carried out using the GenSript codon optimization tool with *E. coli* as the expression host system for the designed vaccine. The server calculates two metrics, such as the codon adaptive index (CAI) and the GC content, essential for measuring the degree of protein translation. The ideal CAI and GC content for efficient protein expression are 1.0 and 30–70%, respectively, beyond which adverse effects on translation and transcriptional efficiencies will be observed. The SnapGene software was utilized for in silico cloning of the codon-optimized vaccine into the pET28 (+). Finally, the recombinant plasmid was subjected to an agarose gel electrophoresis simulation in SnapGene 6.2.1 (https://www.snapgene.com/) (accessed on 12 February 2023) to confirm the in-silico cloning of the constructed vaccine.

### 2.10. In Silico Immune Simulation Analysis

C-ImmSim is an online simulation server that uses a position-specific scoring matrix (PSSM) and machine learning algorithms to anticipate the robustness of the immune response [36]. In this study, three injections were administered at time steps of 1, 84, and 168 (the recommended time between vaccinations), each containing 1000 vaccines without LPS. All other constants were fixed at their default values except “Simulation Steps”, which was defined at 1050.

## 3. Results

### 3.1. Prediction and Prioritization Phase of Potential Peptides

Helper T lymphocytes (HTL) and cytotoxic T lymphocytes (CTL), which bind to MHC class I and class II molecules, are required for the activation of dendritic cells, production of IFN-γ, and triggering apoptosis in tumor cells [17]. Therefore, an ideal vaccine should trigger both HTL and CTL responses to achieve maximum potency. In the present study, a sum of 79 strong binding CTL epitopes were predicted against 12 MHC class I (A1, A2, A3, A24, A26, B7, B8, B27, B39, B44, B58, and B62) super types by NetMHCpan 4.1. Similarly, the NetMHCIIpan-4.0 estimated a total of 36 high-binding HTL epitopes against HLA-DP, HLA-DQ, and HLA-DR. To generate an immunogenic vaccine, the identified epitopes were scrutinized based on the antigenicity, allergenicity, IFN-γ-producing ability, and toxicity. Finally, the top four CTL peptides (AARLRFRCF, ARLRFRCFR, ARLEEHRRV, and REGGFAHAL) (Table 1) and four HTL epitopes (RGRPSTGGGVVRGGR, GGVVRGGRCDVCGKV, RAVLLEHQAVHTGDK, and GQGFVRSARLEEHRR) with the highest antigenic score were considered for the further analysis (Table 2).

B cells play an important role in the tumor microenvironment by inducing humoral immunity and producing immunoglobulins. For these reasons, including B cell epitopes in vaccines could provide added advantages in terms of boosting vaccine-induced CTL antitumor immune responses [37]. In the proposed work, the ABCPred server was used to figure out the 16-mer continuous B cell peptides. Based on the findings, 20 potential B cell epitopes were chosen for further investigation. Out of all selected epitopes, the top four epitopes (PGPEAARLRFRCFRYE, GRPSTGGGVVRGGRCD, SGQIQSPSREGGFAHA, and QVKEESEVTEDSDFLE) were shortlisted based on their non-allergenicity, non-toxicity, and antigenicity profiles (Table 3).

### 3.2. Multi-Epitope Vaccine Engineering

**Figure 1:**
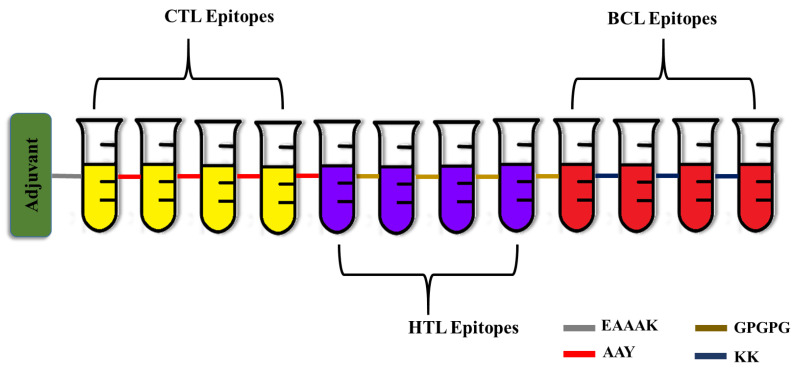
Schematic representation of the engineered vaccine. CTL—Cytotoxic T Lymphocytes; HTL—Helper T Lymphocytes; BCL—Linear B cells.

A robust multi-epitope vaccine was designed using the identified epitopes in fusion with suitable linkers and adjuvants. In most cases, the CTL epitopes are placed immediately before the HTL epitopes. On the other hand, the B cell epitopes are positioned either close to the N-terminal immediately following the EAAAK sequence or adjacent to the C-terminal directly after the CTL and HTL epitopes, but not in between them. Similarly, the adjuvants are positioned either at the beginning of the vaccine peptide sequence or both at the N- and C-termini [38]. To increase immunogenicity and durability, 50S ribosomal L7/L12 protein from Mycobacterium tuberculosis Rv0652 was added to the N-terminus of the vaccine as an immunoadjuvant. The 50S ribosomal L7/L12 protein is a TLR4 agonist, and it enhances the activation of CTL and HTL cells to release IFN-γ via DC maturation and trigger T cell-mediated cytotoxicity [39]. The fundamental advantage of employing linkers is that they enhance antigen processing and presentation, rigidity, and structural flexibility [40]. The linkers KK, AAY, GPGPG, and EAAAK used in the present study are employed widely for peptide vaccine engineering. EAAAK, a rigid linker, was used to fuse the adjuvant, which has the advantage of efficient separation of the functional domains while preserving the functional properties of the epitope. Similarly, the CTL and HTL epitopes were combined by the AAY and GPGPG linkers. The AAY (Ala-Ala-Tyr) linker, being the proteasome’s cleavage spot in mammalian cells, increases the immunogenicity, and GPGPG is a universal spacer that induces TH lymphocyte (HTL) responses, which is crucial for a multi-epitope vaccine. Finally, the B cell epitopes were incorporated using KK linkers, which play a pivotal role in reducing junctional immunogenicity [41]. Altogether the final developed vaccine was 333 residues long and had four CTL epitopes, four HTL epitopes, and four B cell epitopes (Figure 1).

### 3.3. Population Distribution Analysis of the Estimated Epitopes

**Figure 2:**
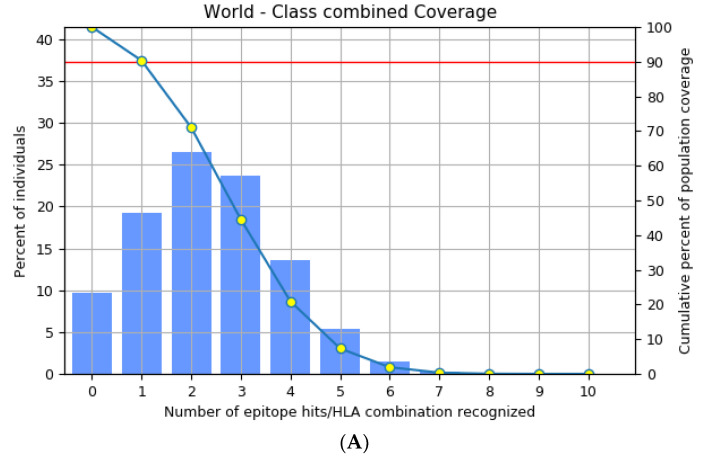

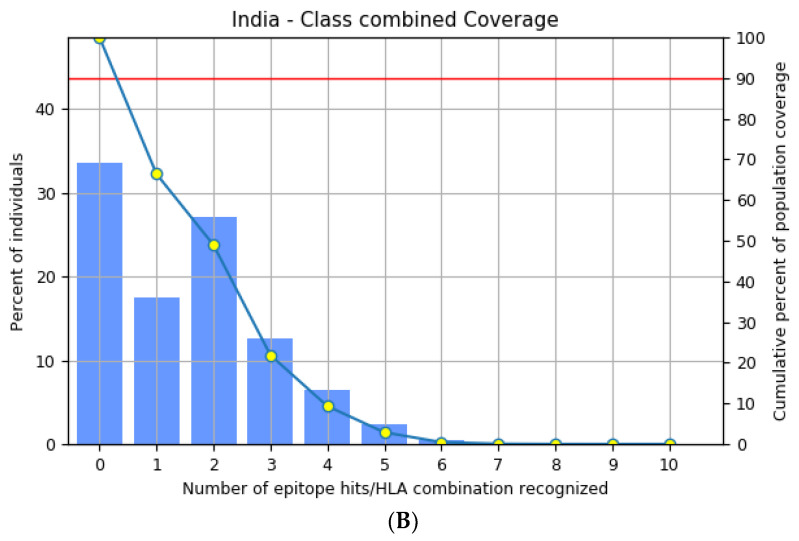
Population frequency coverage analysis report (**A**) globally and (**B**) in India.

The highly polymorphic MHC class I (HLA-A, B, and C) and class II (HLA-DQ, DP, and DR) proteins play significant roles in the adaptive immune system [42]. Thus, developing an effective peptide-based vaccine with epitope affinity for a wide range of allele variants is critical. In this study, we utilized the population coverage tool from the IEDB interface to estimate the combined population frequency of the eight T cell epitopes. Figure 2 reveals that the chosen epitopes cover 90.33% of the world’s population and 75.32% of the Indian population. In a nutshell, the percent population coverage yielded by our proposed vaccine construct indicates that it is effective for the majority of the world’s population.

### 3.4. Assessment of Immunogenic and Physicochemical Properties of the Vaccine

The developed vaccine molecule was examined for a variety of immunogenic attributes. The vaccine was shown to have antigenicity and immunogenicity scores of 0.7833 and 3.720, respectively. Additionally, the vaccine construct was also found to be non-toxic and non-allergic. These findings thus imply that the engineered vaccine molecule is robust and capable of initiating a potent immune response. Furthermore, to gain insight into the efficacy of the vaccine design, the physicochemical properties were computed using the ExPasy ProtParam server. The results indicate that the construct is 333 amino acid residues long with a molecular weight of 350 kDa. The instability index and aliphatic index of the vaccine are 32.80 and 71, respectively. Taken together, the results reflect that the vaccine is highly stable and acidic in nature. Similarly, the theoretical pI that is useful in determining the protein purification methods for the vaccine is found to be 9.37. Additionally, the GRAVY score is found to be −0.376, revealing the hydrophilic behavior of the constructed vaccine. The results are shown in Table 4.

### 3.5. Modelling and Validation of the Secondary and Tertiary Structure

**Figure 3:**
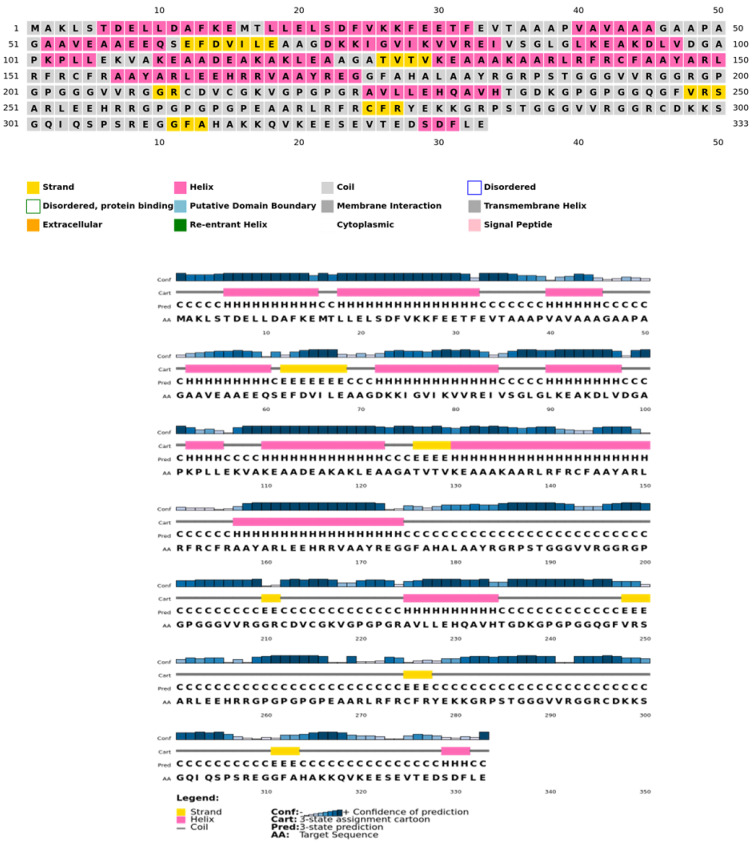
Psipred chart representing the percentage of coils, helices, and sheets in the constructed vaccine.

**Figure 4:**
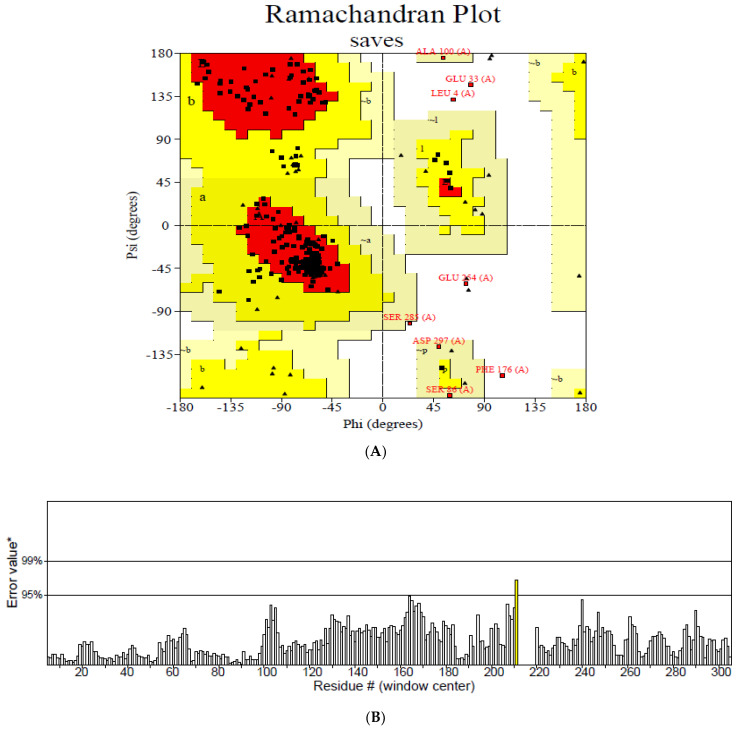

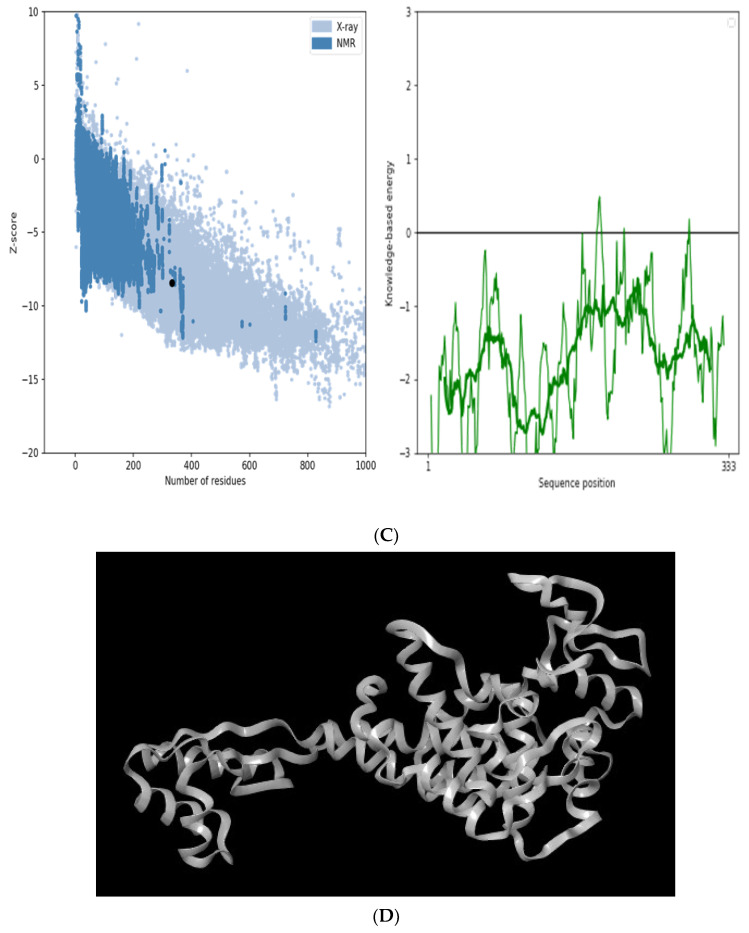
Validation of the modelled vaccine construct (**A**) Ramachandran plot; (**B**) ERRAT graph; (**C**) ProSA graph; (**D**) modelled 3D structure of the vaccine; *—Error value; #—amino acid residues of the protein model.

The secondary structure, which is important for protein folding, is described by the hydrogen bond interactions between carboxyl groups and the amino backbone [43]. The present study utilized the PSIPRED server to anticipate the secondary structure which included 7% of the strand, 39% of the coils, and 54% of the loops (Figure 3). The findings revealed the predominant nature of loops and coils, which highlights the more compact and bonded nature of the proposed vaccine. Furthermore, the three-dimensional structure of the final multi-epitope vaccine was modelled using the ROBETTA server. The stereodynamic quality of the generated five models was validated through ERRAT, PROCHECK, and Pro-SA servers. According to the validation results, Model 1 was adopted for further analysis. The Ramachandran plot reveals that 89% of the amino acids in Model 1 were in the favored domain, validating the structure’s dynamic nature (Figure 4A). Similarly, the non-bonded interactions were assessed using the ERRAT tool, which estimated an overall quality score of 99.683 (Figure 4B). In addition, the Pro-SA server also predicted an overall quality score of −8.44 for our vaccine construct (Figure 4C). Finally, PyMOL was used to visualize the designed vaccine’s three-dimensional structure (Figure 4D).

### 3.6. Identification of Discontinuous B Cell Epitopes

The interactions among conformational B cell regions and antibodies are one of the most significant immunologic mechanisms [44]. A total of six conformational B cell residues with protrusion index scores ranging from 0.59 to 0.78 were computed by the ElliPro server (Table 5), suggesting these regions will be recognized by the antibodies.

### 3.7. Docking Studies of Vaccine with TLR-2, 4, 7, and 9

**Figure 5:**
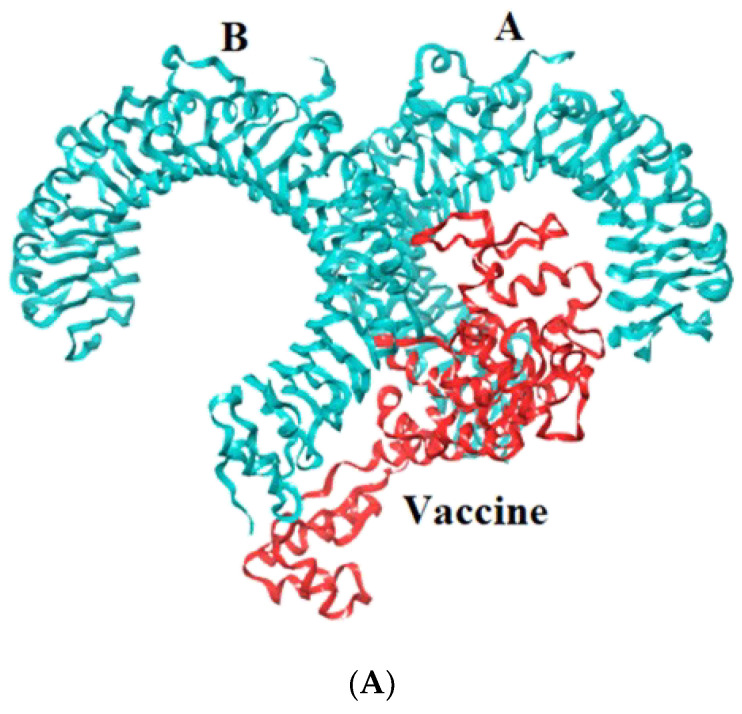

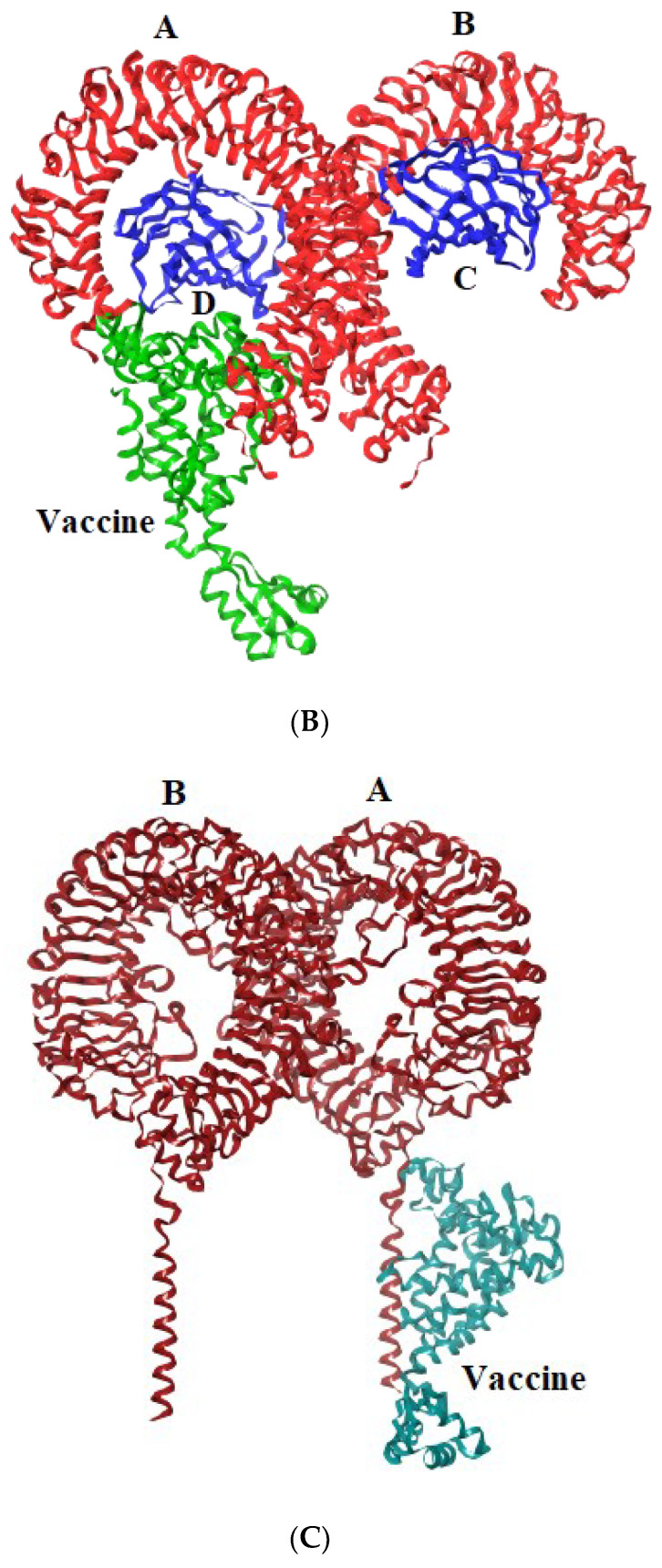

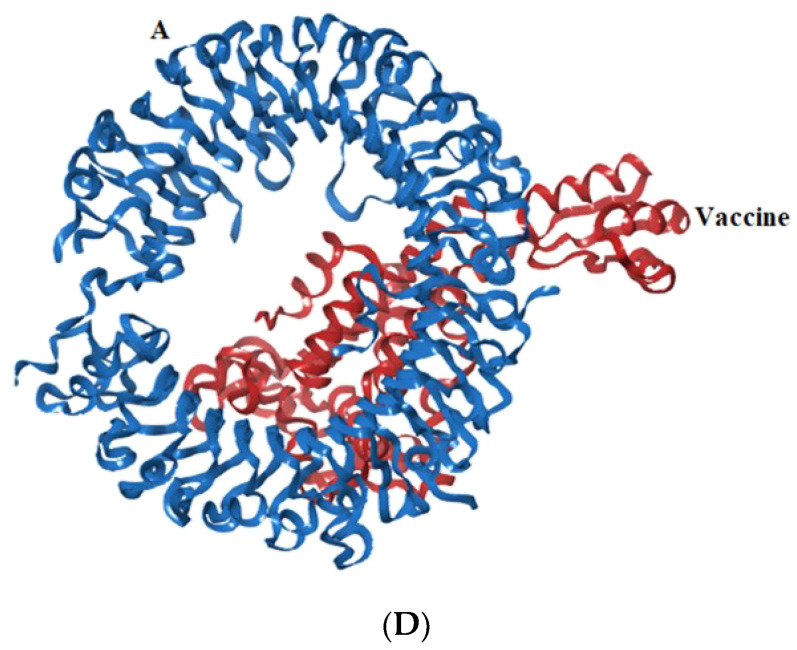
Representation of toll-like receptor and designed vaccine docked complex (**A**) TLR-2 (chain A and B); (**B**) TLR-4 (chain A, B, C and D); (**C**) TLR-7 (chain A and B); (**D**) TLR-9 (chain A).

**Figure 6:**
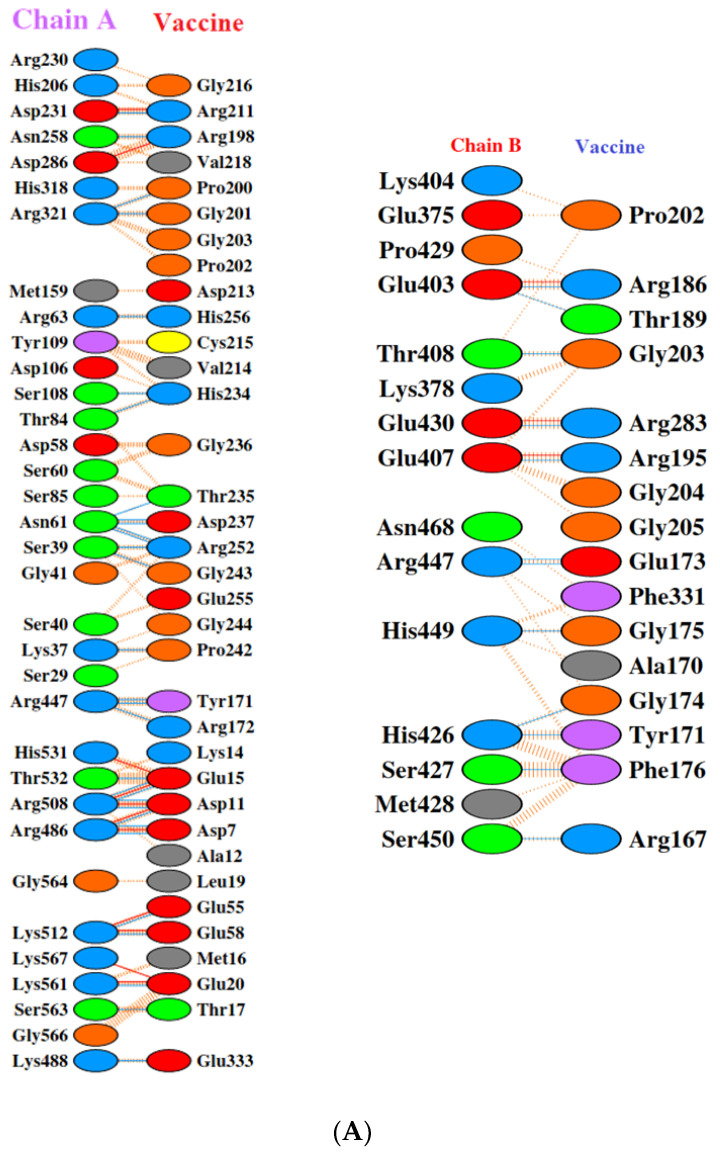

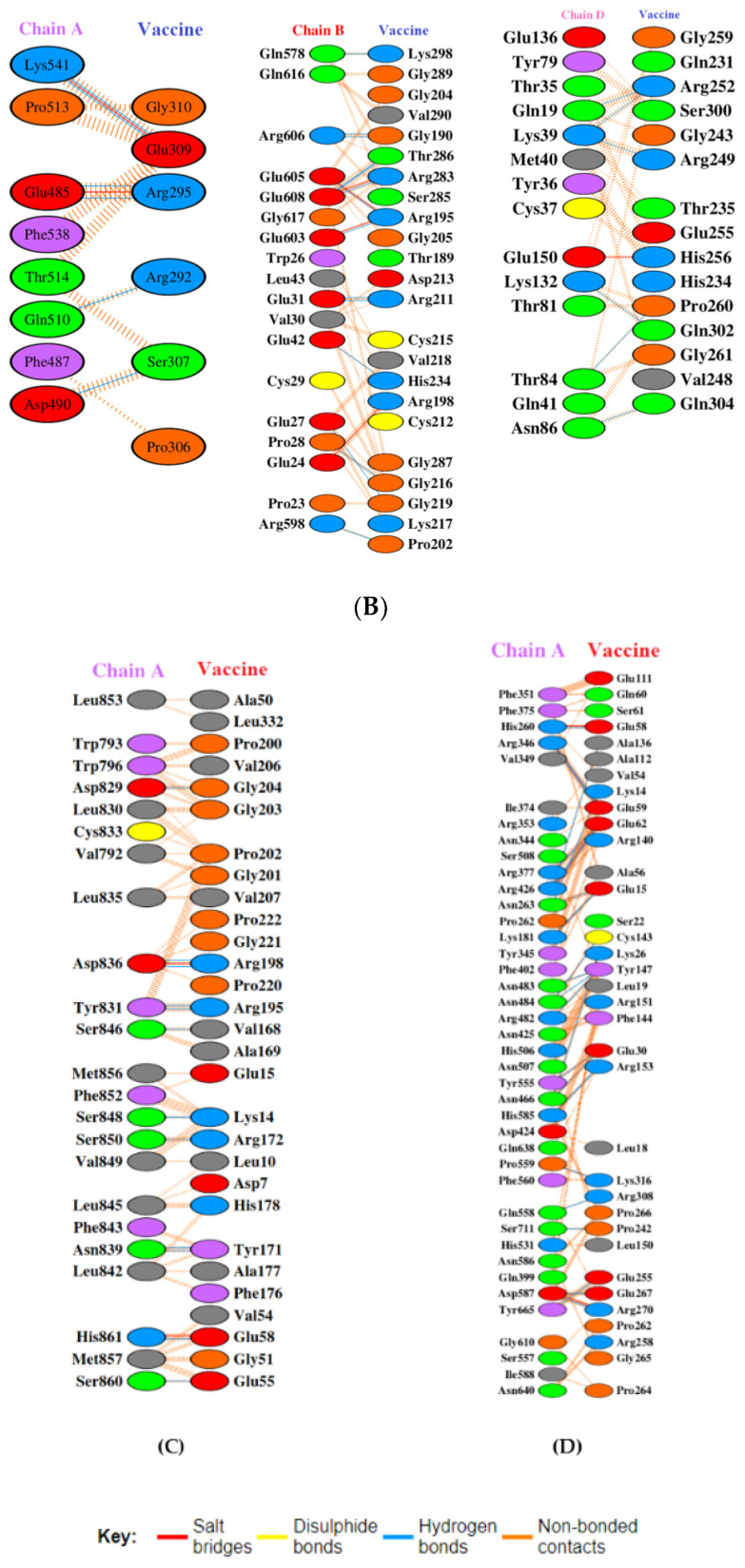
Molecular interaction analysis. (**A**) TLR-2 (chain A, B) with vaccine; (**B**) TLR-4 (chain A, B, and D) with vaccine; (**C**) TLR-7 (chain A) with vaccine; (**D**) TLR-9 (chain A) with vaccine.

Toll-like receptors are immune molecules found on the surface of tumor cells and play a significant role in cancer initiation and evasion. Importantly, they also induce the release of cytokines, including interleukins and interferon-gamma, and aid in antigen recognition. Interestingly, studies suggest that low expression of TLR-2, 4, 7, and 9 aids in immune evasion and tumor formation in TNBC [45]. Moreover, TLR-4- and TLR-9-induced immune responses appear to be the most advantageous for clinical applications [46]. Therefore, to determine the effectiveness of the vaccine’s interaction with immune molecules (TLR-2, 4, 7, and 9), molecular docking analysis was performed using the ClusPro 2.0 server. The binding affinities for TLR 2, 4, 7, and 9 are found to be −929.5 kcal/mol, −1060.3 kcal/mol, −975.6 kcal/mol, and −1091.9 kcal/mol, respectively. The PyMOL software was used to visualize the docked complexes (Figure 5). Furthermore, the PDBSum server was used to identify the molecular interactions between the vaccine and TLRs. In accordance with the results, the vaccine generated a total of 23 hydrogen bonds with TLR-2, 12 with TLR-7, and 26 hydrogen bonds with TLR-4 and 9. In addition to hydrogen bonds, the TLR-vaccine complexes also made salt bridges and non-bonded contacts. The salt bridges and non-bonded contacts also play an essential role in sustaining the protein structure and stability. Overall, the vaccine-TLR-4 (−1060.3 kcal/mol) and vaccine-TLR-9 (−1091.9 kcal/mol) complexes had the lowest binding affinities and the most hydrogen bonds, salt bridges, and non-bonded contacts. This shows that the vaccine has a high level of structural integrity, stability, and affinity for these receptors. Therefore, they were taken into consideration for further validation analysis. The molecular interactions are shown in Figure 6.

### 3.8. Stability Assessment by Molecular Dynamics Simulation

**Figure 7:**
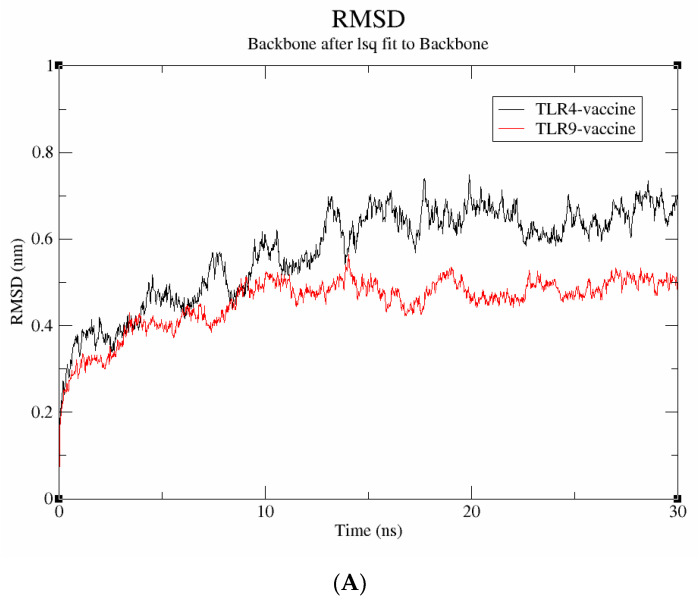

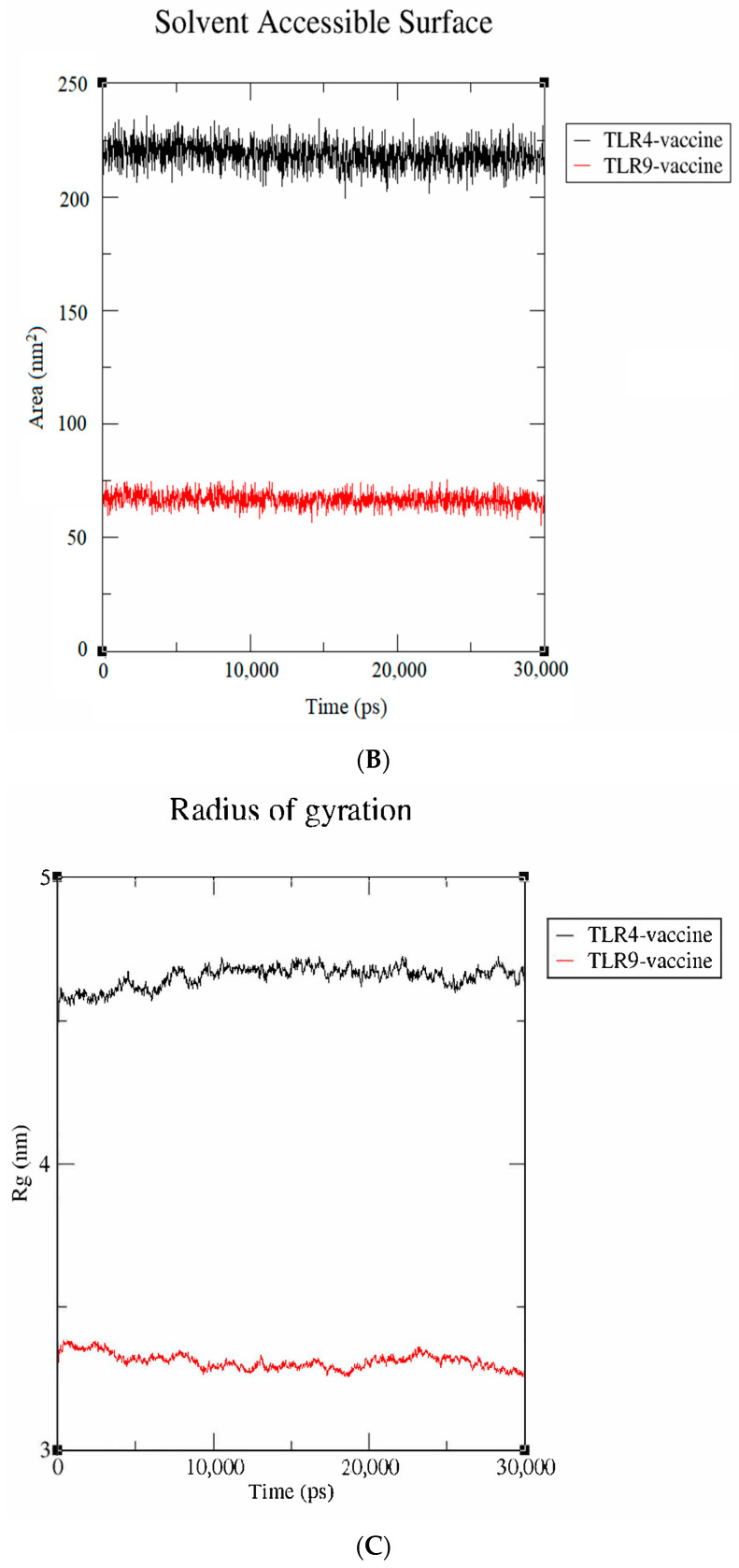
Molecular dynamics simulation studies. (**A**) RMSD; (**B**) SASA; (**C**) radius of gyration.

A molecular dynamics simulation was run for 30 ns to examine the stability and binding mode of the vaccine construct with receptor proteins, including TLR-4 and TLR-9. The data were evaluated using metrics including RMSD, Rg, and SASA to understand more about the stability at the molecular level. The average deviation between the atoms was determined using the RMSD. As seen in Figure 7A, the vaccine-TLR-9 complex has an RMSD score of 0.4 nm and is found to be stable throughout the simulation; in contrast, the TLR-4 vaccine complex has an RMSD value of 0.6 nm and exhibits significant fluctuations throughout the simulation. Therefore, it can be inferred that the vaccine-TLR-9, which exhibited a lower RMSD, has higher structural stability.

The solvent-accessible surface area (SASA) is utilized to compute the protein surface that is exposed to the solvent environment. The SASA values of vaccine-TLR-4 and vaccine-TLR-9 were found to be 80 nm^2^ and 230 nm^2^, respectively (Figure 7B). The result signifies that the TLR-9 complex has a higher SASA, indicating a more stable behavior in a solvent environment. Similarly, the radius of gyration (Rg), which calculates the receptor–ligand flexibility, was also measured. Figure 7C depicts that the vaccine-TLR-9 had a smaller Rg value of 3.1 nm than the vaccine-TLR-4 (4.6 nm), which indicates that the TLR-9 complex remained compact throughout the simulation. Collectively, the results validate that the proposed vaccine had a more stable interaction with the TLR-9 than with the TLR-4 throughout the simulation.

### 3.9. Codon Adaptation and In Silico Cloning of the Vaccine

**Figure 8:**
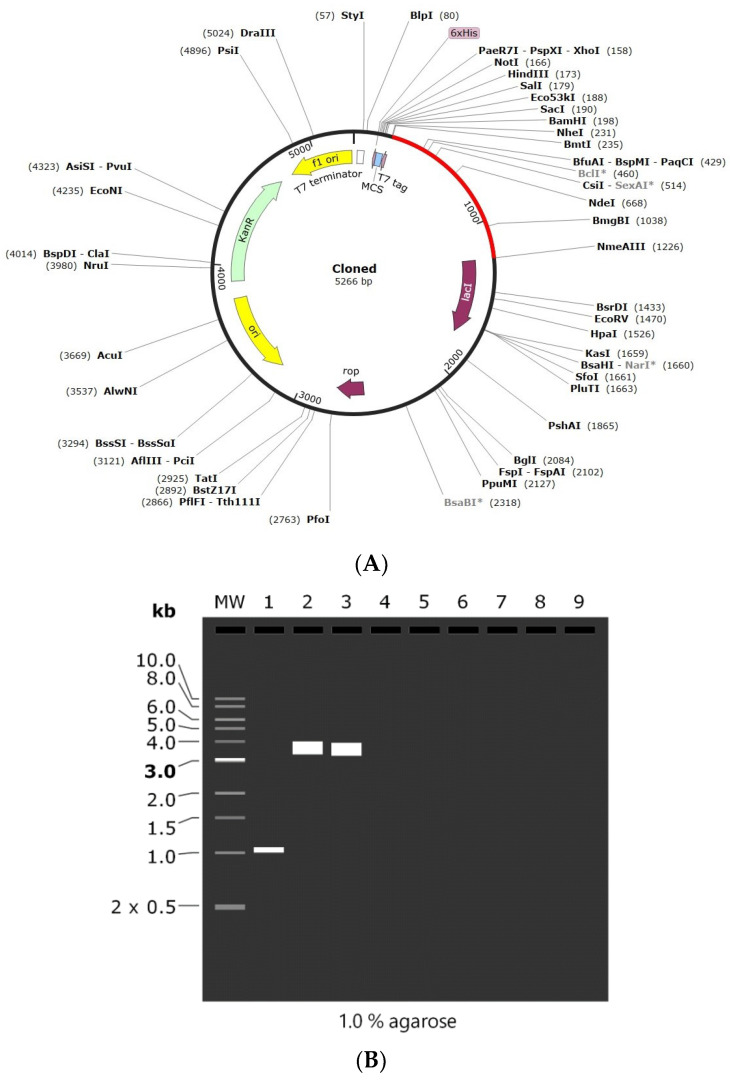
(**A**) In silico cloning of the designed vaccine into pET-28 (+) plasmid. (**B**) 1% Agarose gel electrophoresis simulation where MW: 1 kb DNA ladder, Lane 1: vaccine construct (1011 bp), Lane 2: pET-28a (+) vector (5369 bp), and Lane 3: recombinant plasmid (5266 bp). *—indicated methylation sensitive sites.

Codon adaptation is usually performed to enhance the effectiveness of a target gene’s translation, taking the host organism’s codon bias into account. In the present study, the amino acid sequence was back-translated into a nucleotide sequence and the codons were optimized as per *E. coli* using the GenScript codon optimization tool. The CAI value is observed to be 0.85, which is optimal for the vaccine’s efficient expression in the desired host organism. Similarly, the GC content is also 60.14%, which is within the range of 30–70%, indicating that the vaccine design has strong transcriptional and translational efficiency. Finally, the optimized vaccine was cloned into pET-28(+) vector between the NheI and ApaI restriction sites by SnapGene software. The length of the final cloned vector was found to be 5266 bp (Figure 8A). Finally, agarose gel simulation was performed to confirm the cloned product (Figure 8B). The simulation was conducted at a concentration of 1%, which is an optimal experimental concentration for plasmids larger than 5 kB, and Tris-borate-EDTA (TBE) was chosen as the buffer solution due to its better buffering capabilities [47,48]. These results demonstrate that the intended vaccine was successfully cloned into the plasmid.

### 3.10. Immune Simulation Studies

**Figure 9:**
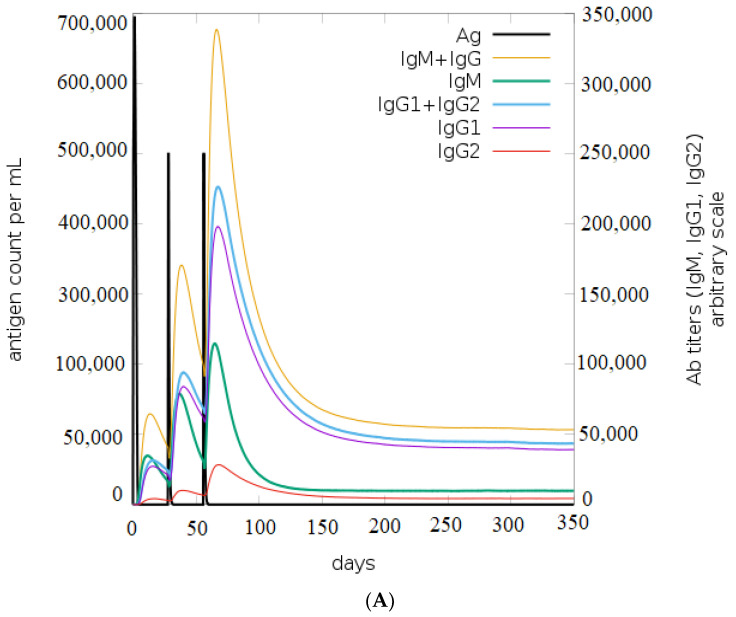

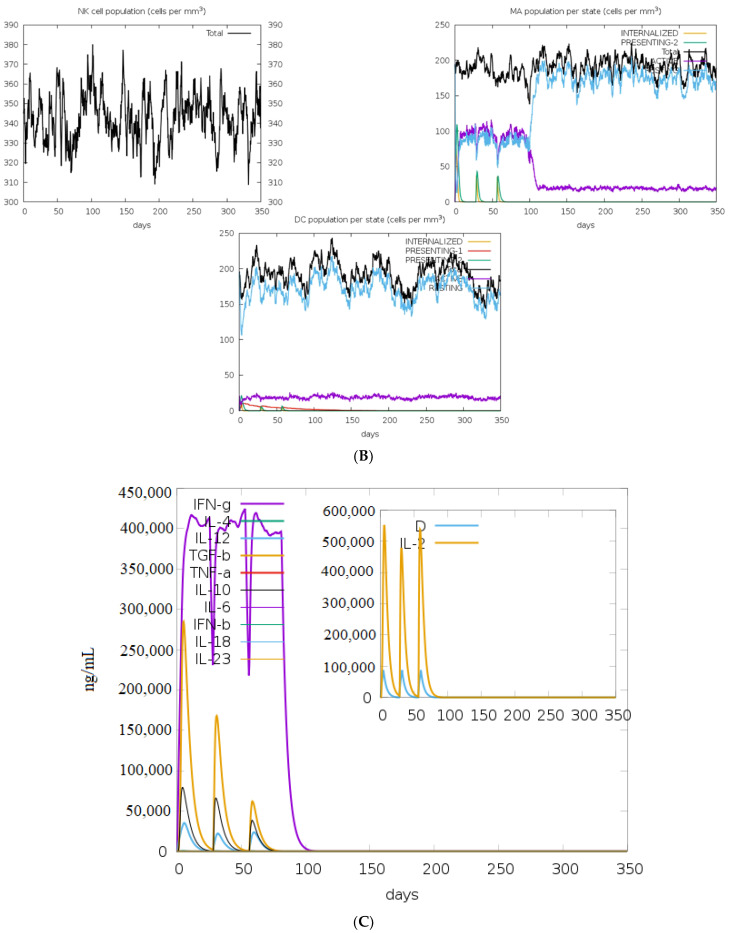
In silico immune simulation analysis. (**A**) Level of immunoglobulins after immunization; (**B**) levels of immune cells after vaccination; (**C**) level of cytokines and interleukins after immune response.

Based on the results of the C-ImmSim, it can be seen that the secondary and tertiary responses were better than the primary response. A considerable spike in the levels of IgM and IgG antibodies was observed after the second and third injections, followed by a decrease in the level of the antigen (Figure 9A). Similarly, it is evident from Figure 9B that the levels of NK cells, macrophages, and DC cells have distinctly increased. As a result, higher levels of IL-2 and INF-gamma were detected (Figure 9C). This shows that our engineered vaccine would cause a strong immune response that would help fight against TNBC.

## 4. Discussion

Authors should discuss the results and how they can be interpreted from the perspective of previous studies and of the working hypotheses. The findings and their implications should be discussed in the broadest context possible. Future research directions may also be highlighted.

## 5. Conclusions

TNBC’s major concerns are its high mortality rate, recurrence, and limited therapeutic options. In the present study, an ensemble of immunoinformatics approaches was utilized to design a novel chimeric subunit vaccine against metastatic TNBC. Initially, the immunogenic peptides that are non-toxic and non-allergic were identified from MZF-1 protein. The modelled three-dimensional structure of the vaccine demonstrated that it efficiently interacts with toll-like receptors (TLR-4 and TLR-9) and thereby induces strong cellular responses. The vaccine-TLR complex stability was validated through molecular dynamic simulation analysis. The results revealed that the engineered vaccine candidate had a lower rmsd and Rg value, reflecting the molecules’ structural integrity and compact nature. Similarly, the construct had higher SASA, indicating its stability in the solvent atmosphere. Finally, the immunological simulation studies also demonstrated the vaccine’s ability to trigger a host immune response. Overall, our study’s findings highlight that the designed multi-epitope vaccine could be used as a treatment for metastatic triple-negative breast cancer. However, to confirm its efficacy, additional experimental validations are required.

## Figures and Tables

**Table 1:** Predicted MHC I binding epitopes and their immunogenic properties.

S. No	Peptides	MHC I Alleles	Vaxijen Score	Allergenicity	Toxicity
1	AARLRFRCF	HLA-B*08:01	1.2291	Non-allergen	Non-toxic
2	ARLRFRCFR	HLA-B*27:05	1.3903	Non-allergen	Non-toxic
3	ARLEEHRRV	HLA-B*27:05	1.0612	Non-allergen	Non-toxic
4	REGGFAHAL	HLA-B*40:01	1.3054	Non-allergen	Non-toxic

HLA—Human Leukocyte Antigen, *—indicates that HLA typing and its corresponding location in chromosome.

**Table 2:** Predicted MHC II binding epitopes and their immunogenic properties.

S. No	Peptides	MHC II Alleles	Vaxijen Score	Allergenicity	Toxicity	IFN-γ Inducers
1	RGRPSTGGGVVRGGR	HLA-DQA10401, HLA-DQB10301, HLA-DQA10501, HLA-DQA10505	1.1247	Non-allergen	Non-toxic	Inducer
2	GGVVRGGRCDVCGKV	HLA-DQB10301, HLA-DQA10501, HLA-DQA10505	1.416	Non-allergen	Non-toxic	Inducer
3	RAVLLEHQAVHTGDK	HLA-DRB1*0103	0.8254	Non-allergen	Non-toxic	Inducer
4	GQGFVRSARLEEHRR	HLA-DRB1*1401, HLA-DRB1*1402, HLA-DRB1*1454, HLA-DRB1*0801, HLA-DRB1*0803, HLA-DRB1*1101, HLA-DRB1*1302, HLA-DRB1*1303	0.5788	Non-allergen	Non-toxic	Inducer

HLA—Human Leukocyte Antigen, *—indicates that HLA typing and its corresponding location in chromosome.

**Table 3:** Predicted B cell epitopes and their characteristics.

S. No	Peptides	Vaxijen Score	Allergenicity	Toxicity
1	PGPEAARLRFRCFRYE	1.2090	Non-allergen	Non-toxic
2	GRPSTGGGVVRGGRCD	1.4314	Non-allergen	Non-toxic
3	SGQIQSPSREGGFAHA	1.1019	Non-allergen	Non-toxic
4	QVKEESEVTEDSDFLE	1.2340	Non-allergen	Non-toxic

**Table 4:** Physicochemical characteristics of the constructed vaccine.

S. No	Parameters	Score
1	Immunogenicity	3.72024
2	Antigenicity	0.7833
3	Molecular weight	350 kDa
4	Theoretical pI	9.37
5	No. of amino acids	333
6	Instability index	32.80
7	Aliphatic index	71.62
8	GRAVY	−0.376

GRAVY—Grand average of hydropathicity index; Theoritical pI—Isoelectric point.

**Table 5:** Predicted discontinuous B cell epitopes.

S. No	Residues	Number of Residues	Score
1	ILEAAGDKKIGVIKVVREIVSGLGLKEAKDLVDGAPKPLLVAKEAADEAKAKLEAAGATVTV	62	0.789
2	RRGPGPGPGPEA	12	0.721
3	GPSTGGGRGGRCDKKSGQQSPSREGGFAH	31	0.673
4	MAKLSEPSTGGGVVRGGRGPGPGGGVVRGGRCDVCGKVGPGPGHAVHTGDKGPGPGGQG	59	0.664
5	DKKET	5	0.641
6	EMTLE	5	0.598

## Data Availability

Not applicable.
